# Pacing Electrocardiogram Detection With Memory-Based Autoencoder and Metric Learning

**DOI:** 10.3389/fphys.2021.727210

**Published:** 2021-12-17

**Authors:** Zhaoyang Ge, Huiqing Cheng, Zhuang Tong, Lihong Yang, Bing Zhou, Zongmin Wang

**Affiliations:** ^1^School of Information Engineering, Zhengzhou University, Zhengzhou, China; ^2^Cooperative Innovation Center of Internet Healthcare, Zhengzhou University, Zhengzhou, China; ^3^Big Data Center of Clinical Medicine, The First Affiliated Hospital of Zhengzhou University, Zhengzhou, China; ^4^Department of Cardio-Pulmonary Function, Henan Provincial People's Hospital, Zhengzhou, China

**Keywords:** electrocardiogram signals, autoencoder, heartbeat arrhythmias detection, metric learning, attention mechanism

## Abstract

Remote ECG diagnosis has been widely used in the clinical ECG workflow. Especially for patients with pacemaker, in the limited information of patient's medical history, doctors need to determine whether the patient is wearing a pacemaker and also diagnose other abnormalities. An automatic detection pacing ECG method can help cardiologists reduce the workload and the rates of misdiagnosis. In this paper, we propose a novel autoencoder framework that can detect the pacing ECG from the remote ECG. First, we design a memory module in the traditional autoencoder. The memory module is to record and query the typical features of the training pacing ECG type. The framework does not directly feed features of the encoder into the decoder but uses the features to retrieve the most relevant items in the memory module. In the training process, the memory items are updated to represent the latent features of the input pacing ECG. In the detection process, the reconstruction data of the decoder is obtained by the fusion features in the memory module. Therefore, the reconstructed data of the decoder tends to be close to the pacing ECG. Meanwhile, we introduce an objective function based on the idea of metric learning. In the context of pacing ECG detection, comparing the error of objective function of the input data and reconstructed data can be used as an indicator of detection. According to the objective function, if the input data does not belong to pacing ECG, the objective function may get a large error. Furthermore, we introduce a new database named the pacing ECG database including 800 patients with a total of 8,000 heartbeats. Experimental results demonstrate that our method achieves an average F1-score of 0.918. To further validate the generalization of the proposed method, we also experiment on a widely used MIT-BIH arrhythmia database.

## 1. Introduction

The electrocardiogram (ECG) is an important tool in the everyday practice of clinical medicine (Hannun et al., 2019), especially for patients who are fitted with a pacemaker. The application of a pacemaker effectively alleviates the condition of patients with heart disease and extends the survival period of patients. But these patients require regular in-hospital checks of the pacemaker and cardiac abnormalities. Therefore, remote cardiac monitoring for pacemaker patients becomes increasingly important. To find cardiac abnormalities in time, computer-aided diagnosis provides real-time ECG analysis without any manual intervention by physicians.

The pacing ECG detection still confronts many challenges. First, the procedure of remote ECG diagnosis only contains ECG signals from the patients, and doctors cannot check the patient's medical history in advance. Second, there are different performances in morphological features between pacing ECG and routine ECG for the same disease. And in clinical ECG data, the ECG morphology of the pacemaker patient is interfered with by the pacemaker, which brings disturbance to the ECG abnormality diagnosis. For example, ventricular pacing is often confused with the left bundle branch block of routine ECG. Suppose we do not inform the doctor that the remote ECG data comes from a pacemaker patient. In that case, the doctor may diagnose certain pacing heart rhythms as other abnormalities of the routine ECG. To solve the above problems, we constructed an extensive, novel ECG database named Pacing ECG Database, which includes 800 samples of ECG data annotated by the clinician. To the best of our knowledge, this is the first ECG signals database faced with pacing ECG. The automatic recognition of the pacemaker provides a solid guide to distinguish abnormal ECG accurately, which can improve the performance of abnormal ECG classification.

Motivated by the recent success of autoencoder as a time series detection tool, several promising autoencoder for ECG abnormality detection have been proposed. Specifically, it is generally assumed that the reconstruction error trained by the traditional autoencoder will be lower for the training data of the input type. In contrast, reconstruction error becomes significant for other abnormalities. Therefore, the autoencoder (AE) is trained by minimizing the reconstruction error in each class and then utilizes an objective function based on reconstruction error to classify ECG abnormalities. However, many works have proved that autoencoder's generalization ability can sometimes well express other abnormal samples that the inputs do not belong to the training type (Zong et al., 2018; Gong et al., 2019). This is because the decoder is powerful for decoding some abnormal encodings well, so the AE sometimes also gets a lower reconstruction error for other ECG abnormalities.

**Figure 1:**
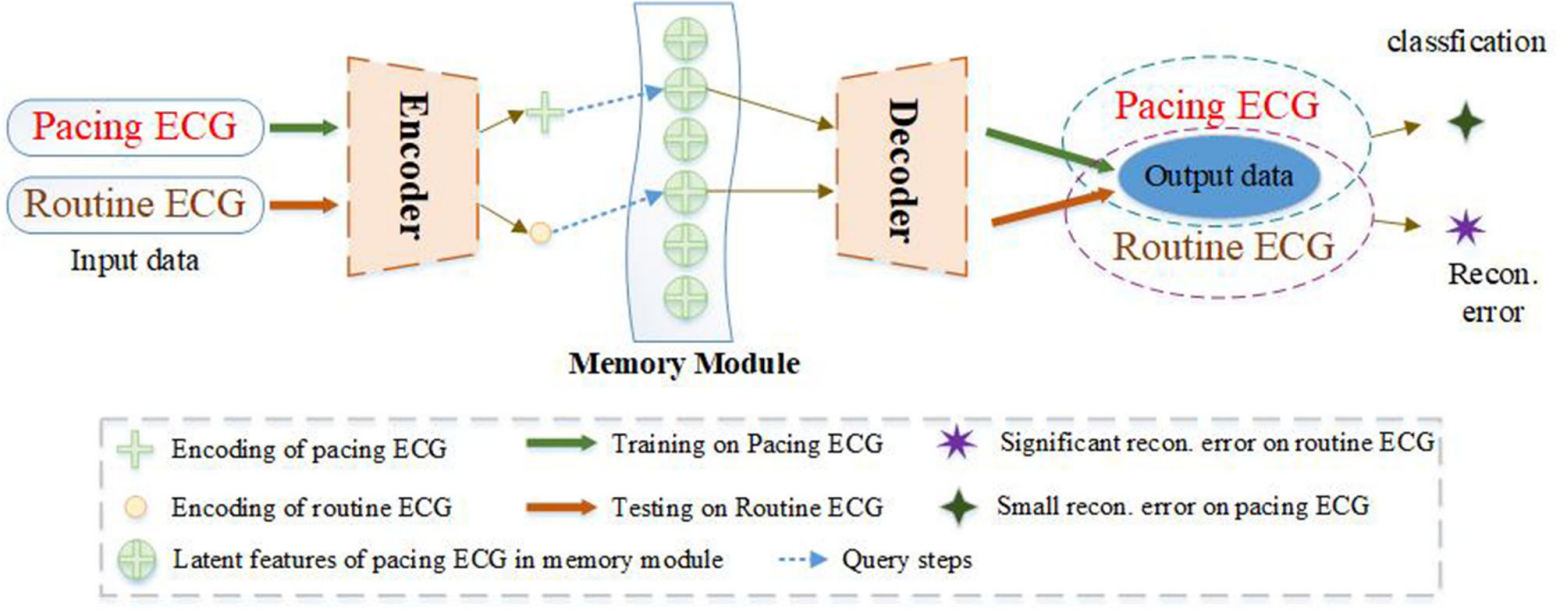
The ECG abnormality detection via the proposed MAE. A sample of training on the pacing ECG database, the memory module records the latent features of the corresponding training ECG type. Given an input sample of other types, the MAE queries the most correlation latent features in the memory module for data reconstruction, resulting in an output significantly different from the input data. But if the input sample belongs to the training type. The MAE can reconstruct very well according to the memory module.

To improve the drawback of traditional AEs, we propose a new model of memory-based autoencoder (MAE) for pacing ECG detection, as illustrated in Figure 1. In the MAE model, we first utilize a deep autoencoder to extract the typical features of high-dimensional ECG data. But the decoder of MAE does not directly reconstruct the data from the encoding. We added a memory module between the encoder and decoder. The memory module is to record and query the typical features of the training pacing ECG type. MAE does not directly feed features of encoder into the decoder but uses the features to retrieve the most relevant items in memory module. Those features are aggregated and delivered to the decoder. Meanwhile, we further utilize a sparse coding strategy to induce sparsity for the memory module, which can easily match the memory items to the query of the feature space.

We are also inspired by metric learning. The goal of metric learning is to learn a distance metric that puts the same positive types close together and negative types far away. In this paper, the MAE is trained by minimizing the error of objective function on the pacing ECG and then uses the error of objective function as an indicator of pacing ECG detection. In the training process, the features of the pacing ECG are learned and stored by the MAE model. The purpose is to obtain a lower reconstruction error for pacing ECG. In the detection process, the reconstruction data of the decoder is fused with the features in the memory module. Because the reconstruction data is obtained from the feature of the pacing ECG in the memory module, the output of the decoder tends to be close to the pacing ECG. In the results, if the input data does not belong to pacing ECG, the objective function may get a significant error. The proposed MAE is not only for detecting the pacing ECG but also can be applied to solve other ECG abnormality detection. We also apply the proposed MAE on the MIT-BIH Arrhythmia Database. The experiments prove the excellent generalization and effectiveness of the model.

To summarize, the contributions of this paper are as follows.

(1) A novel autoencoder framework named MAE is proposed to detect the pacing ECG. The memory module is added between the encoder and decoder. The memory module is used to record the features of the training data.
(2) We introduce a new objective function that is based on metric learning, which can better represent the error among the different types. Because of the memory module, the reconstruction tends to be close to the training types. When the input types are not similar to the training types, the objective function has a significant score.
(3) We collect a new database named Pacing ECG Database for evaluation of the MAE framework. The database includes 800 annotated samples and each ECG data is de-identified according to the privacy policy.
(4) The experiments conducted on Pacing ECG Database, demonstrate that the proposed MAE reliably improves the performance of pacing ECG. To further validate the generalization, the MAE framework is also applied on MIT-BIH Arrhythmia Database and is superior to the state-of-the-art detectors.

This reminder of the paper is organized as follows. Section 2 presents the related works. Section 3 introduces the proposed memory-based autoencoder method. The experimental results and analysis are given in section 4. Finally, section 5 concludes this paper.

## 2. Related Work

In recent years, many approaches have been proposed to automatically process physiological signals in the field of artificial intelligence (Gao et al., 2018; Wang et al., 2020; Yao et al., 2020; Zhou and Tan, 2020). For abnormal ECG detection, the performance is consistently improved in terms of accuracy on major challenges and benchmarks, such as MIT-BIH (Moody and Mark, 2001), CPSC_2018 (Liu et al., 2018a). Nevertheless, there are few solutions for pacing ECG, which pacing ECG is only regarded as a kind of ECG abnormality in the classification task. In this paper, we focus on improving the accuracy detection of pacing ECG.

### 2.1. ECG Abnormality Classification

Cardiovascular diseases can be divided into cardiomyopathy, ischemic heart disease, myocardial infarction and so on (Hao et al., 2021). Many clinicians focused on using computer-aided diagnosis to detect one of the heart diseases (Baloglu et al., 2019). For example, Adam et al. (2018) focused on classifying hypertrophic heart disease, dilated cardiomyopathy, hypertrophic cardiomyopathy. Many researchers are interested in studying ECG bundle branch block, which is heart disease with high mortality. It can be divided into the left bundle branch block (LBBB) and the right bundle branch block (RBBB) (Zhang et al., 2012). There is also a part of clinical research that focused on the use of electrocardiograms to detect specific abnormalities such as myocardial infarction (Liu et al., 2018b; Baloglu et al., 2019).

Some approaches have been devoted to improving the performance of ECG abnormality diagnosis in recent years (Mondéjar-Guerra et al., 2019; Hao et al., 2021; Wang et al., 2021). The signal processing is essential for clinical monitoring. Typically, one kind of methods (Mondéjar-Guerra et al., 2019; Wang et al., 2021) is to diagnose ECG on features of high-dimensional space with rich fine features of ECG abnormalities. In previous studies on ECG abnormality classification, which focused on preprocessing to segment the raw ECG sequence to heartbeats (Sodmann and Vollmer, 2020). And then, feature descriptions of abnormal ECG are calculated the from the heartbeats (Sangaiah et al., 2020), for example, RR interval features extraction (Chen et al., 2017), wavelets (Mar et al., 2011; He et al., 2018), higher-order statistics (HOS) (Osowski and Tran, 2001). Other methods based on deep learning can learn useful features from raw ECG data without requiring signal preprocessing (Fan et al., 2018; Ma et al., 2020). A series of typical strategies focus on designing network architecture to extract multiple features to improve ECG abnormality classification accuracy. However, in practice, these methods need a sufficient amount of handcraft labels or features.

Recent studies of ECG abnormality classification have concentrated on deep learning (Hannun et al., 2019; Saadatnejad et al., 2020; Zhang et al., 2020). A convolutional neural network (CNN) is an effective method for extracting features due to its local connectivity and parameter sharing. Hannun et al. (2019) developed a 34-layer CNN that classifies 12 types of ECG signals and achieves cardiologist-level performance. The RNN-based (Wang et al., 2018; Chen et al., 2020) method, such as the Gated Recurrent Unit (Zhang et al., 2019), the Long Short Term Memory (Tan et al., 2018; Saadatnejad et al., 2020), is a type of neural network used for processing ECG signal. The RNN is used to extract global features and then classified the ECG abnormalities. Saadatnejad et al. (2020) proposed a continuous and real-time patient-specific ECG classification algorithm based on wavelet transform and multiple LSTM. Other effective methods (Chen et al., 2020; Wang et al., 2020; Yao et al., 2020) to develop the architectures combining CNN with RNN for detecting multi-class ECG abnormalities. But in these works of ECG diagnosis, the pacing rhythm is only detected as a kind of ECG abnormality.

### 2.2. Autoencoder

The autoencoder belongs to unsupervised tasks of deep learning and does not need data annotation for training samples. The autoencoder is composed of three layers, in which the number of neurons in the input layer is the same as the number of neurons in the output layer, and the number of neurons in the middle layer is less than that of the input and output layer. During the training phase, for each training sample, a new signal will be generated in the output layer through the network. The purpose of training is to make the output signal and the input signal as similar as possible. In the testing phase of autoencoder, it can be composed of two parts. The first part is the input layer and the middle layer, which can use to compress the signal. The second part is the middle layer and the output layer, which can restore the compression signal.

With the development of artificial intelligence, it is already widely applied to many areas such as bioinformatics (Oyetunde et al., 2018), engineering technology (Samaniego et al., 2020) and clinical medicine (Chen et al., 2018). Thinsungnoen et al. (2018) proposed the deep autoencoder (AE) which is a powerful tool to deal with the high-dimensional data in the unsupervised task of processing ECG signals. They have great success in some application domains as well, such as denoising autoencoders (DAEs) (Dasan and Panneerselvam, 2021), ECG data dimension reduction (Wang et al., 2013). And a series of work has been conducted in ECG data classification using an autoencoder model. However, in practice, the pacemaker can interfere with the ECG signal. It often leads to the morphological difference between cases with pacemaker and cases without pacemaker in the same ECG abnormality. Lack of pacing ECG data has limited many models design for abnormal ECG classification. Meanwhile, the existing autoencoder for ECG classification algorithms still has a misdiagnosis rate. Therefore, we collect a large pacing ECG database and design a novel autoencoder model to detect the pacing ECG.

Traditional autoencoders are mainly used for ECG signal reconstruction. For example, Majumdar et al. (2016) design a stacked autoencoder (SAE) model which mainly uses semi-supervised deep learning approach for ECG signal reconstruction. In the research of industrial anomaly detection, Hasan et al. (2016) use the reconstruction error of a convolutional AE to detect the anomalies in video sequences. However, these methods neglect the generalization capability of the autoencoder model and lack a mechanism to encourage the autoencoders to produce larger reconstruction errors for abnormalities.

Recently, existing a novel method introduces the memory-augmented networks to solve the anomaly detection by reconstructing the input data (Kim et al., 2018). Gong et al. (2019) detect the anomalies according to the reconstruction error of a memory-augmented AE. The memory module can record features stably. Santoro et al. (2016) use the idea to handle the one-shot learning problem. These methods show significant performance gain, especially for anomaly detection. However, these algorithms only detect one class, which makes them infeasible for ECG diagnosis.

The previous work focused on the autoencoder conduct to deal with the issue of data imbalanced or noise reduction. Inspired by these methods, we propose the MAE model using an attention-based memory module to record latent features of corresponding ECG abnormalities. We also propose a quantitative assessment criterion to cluster each ECG abnormality type. Then, we verify the performance of our model on different databases. The proposed MAE model shows drastic improvements for ECG abnormality diagnosis.

## 3. Method

**Figure 2:**
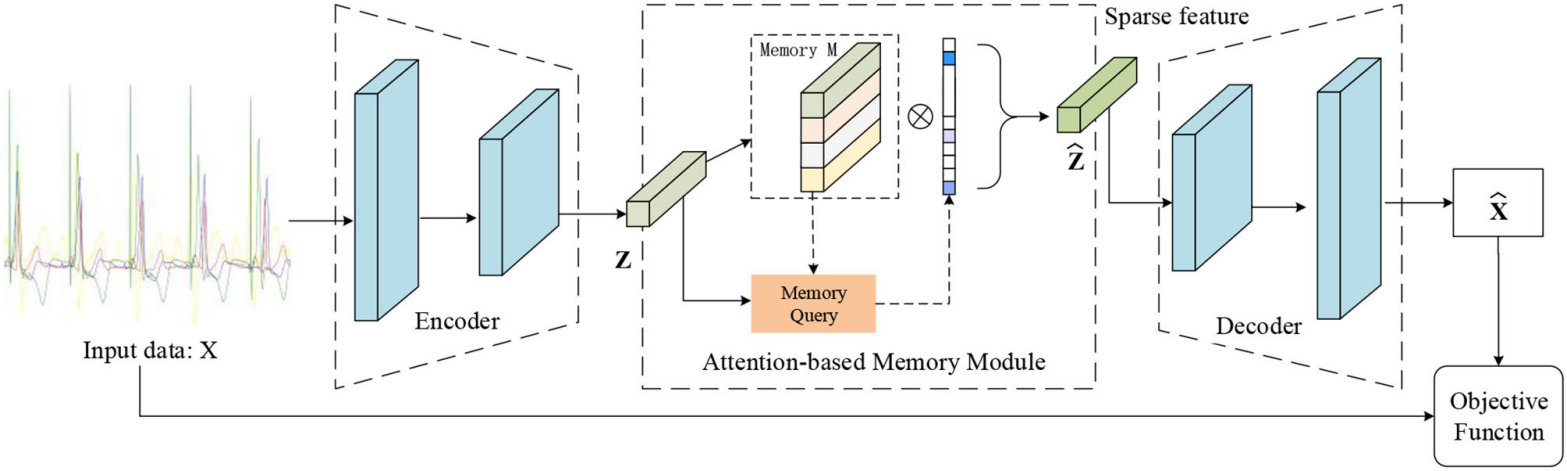
Illustration of the proposed MAE model. The memory module records latent features and the memory query can obtain the weight of the degree of similarity between the features of input data and the record features. Note that the output of the memory module is the input of the decoder.

Previous AE architectures for ECG signal processing focused on data denoising and data dimensionality reduction. In this paper, we propose a novel autoencoder architecture containing a memory module that can record the latent features of the training type, as shown in Figure 2. And we also introduce a new objective function that can calculate a similarity score between the output of the decoder and the input data. As a result, we define the estimated score of the data as the clustering criteria. This makes the proposed MAE model especially suitable for ECG abnormality classification.

### 3.1. Encoder and Decoder

The encoder of MAE model can obtain the features from input data, which is beneficial for data dimension reduction. The features can be used as a key to match the relevant features in the memory module. In our method, the output of the encoder can be seen as a generator of a feature dictionary. The decoder is trained to reconstruct the samples by taking the retrieved memories as input.

In this paper, we consider multi-channel time-series recordings of ECG. We first define *X* to represent the domain of the ECG data samples. Each ECG is a multivariate time-series where the rows define the channel dimension, and the columns capture the time dimension. One ECG sample is represented by the following matrix $x_{i}=\left\{x_{i}^{1},x_{i}^{2},...,x_{i}^{C}\right\}\in X^{C\times T}$ where *C* denotes the channels of ECG sequence and *T* is the number of sample points per channel. Our MAE architecture is first composed of an encoder, where *Z* represents the domain of the encodings. Let *f*_*e*_(*X*) → *Z* denote the encoder. The encoder aims to provide a low dimensional latent representation domain *Z* from the input data domain *X*. Given a sample *x*_*i*_ ∈ *X*, the encoder converts it to an encoded representation as *z*_*i*_ ∈ *Z*, as follows:


(1) $$\begin{array}{r} z_{i}=f_{e}\left(x_{i};\theta_{e}\right), \end{array}$$

where θ_*e*_ denotes the parameters of the encoder *f*_*e*_.

The second half of the MAE model architecture contains a decoder, which aims to reconstruct the samples. Let *f*_*d*_(*Z*) → *X* denote the decoder. And the decoder is trained to reversely mapping a latent representation $\hat{z_{i}}\in Z$ to the domain *X*. It should be specially explained that the proposed MAE model is different from the standard AE model. For tradition AE model, the $\hat{z_{i}}$ should be *z*_*i*_. But in this paper, the latent features *z*_*i*_ in the training phase are stored in the feature dictionary, and in the testing phase are regarded as a keyword to query, and $\hat{z_{i}}$ is obtained from the memory unit, as follows:


(2) $$\begin{array}{r} \hat{x_{i}}=f_{d}\left(\hat{z_{i}};\theta_{d}\right), \end{array}$$

where θ_*d*_ denotes the parameters of the decoder *f*_*d*_.

**Figure 3:**
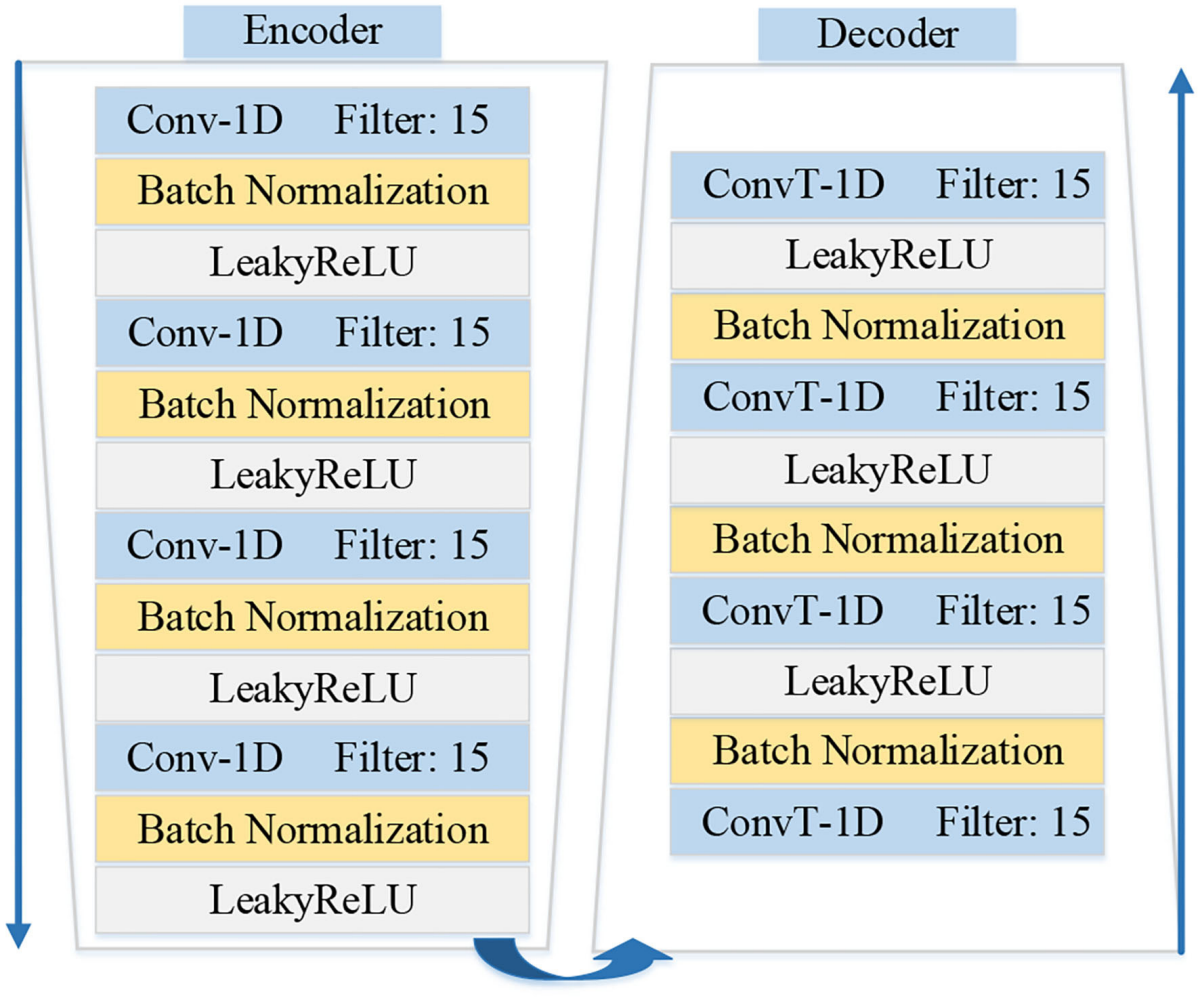
Illustration shows the details architectures of encoder *f*_*e*_ and decoder *f*_*d*_ in the MAE model.

The architectures of encoder *f*_*e*_ and decoder *f*_*d*_ are shown in Figure 3. The architecture of the encoder contains four one-dimensional convolutional layers. And each convolutional layer is followed by a normalization layer and an activation layer. The convolution layers with the kernel size of 1 × 15 are applied to capture the latent features. The fractionally-strided convolution is used in the decoder, which is often used to enlarge the size of the image in image processing. The operation of the fractionally-strided convolution and the normal convolution is exactly the opposite. In this paper, we use the fractionally-strided convolution to restore the input ECG signal type from the low-dimensional latent features.

### 3.2. Attention-Based Memory Module

The purpose of the memory module is to record the most representative features in the input pacing data during training. The above section encoder converts the input data to the internal feature representation. The memory module can be regarded as a dictionary *D* with a querying scheme and is designed as a matrix *D* ∈ *R*^*N*×*C*^ containing *N* real-valued vectors of fixed dimension and to record the prototypical correlation internal feature of pacing ECG during training. The output feature map ẑ of memory unit combines the new input *z* with the entry in current memory state *k*, where *k* ∈ *R*^1×*N*^ is a row vector with non-negative entries that sum to one. The weight vector *k* is computed according to *z*. The output of latent features ẑ will be obtained via ẑ = *k* · *D*.

Let the row vector *m*_*i*_, ∀*i* ∈ {1, 2, …, *N*} denote the *i*−*th* row of *D*, where {*N*} denotes the set of integers from 1 to N. Each *m*_*i*_ denotes the item in the dictionary *D*. The parameter *N* defines the maximum capacity of the memory unit. The typical memory module is developed to query prototypical pacing ECG features, as illustrated in Figure 2.

To be specific, we first introduce a query strategy that computes attention weights *k*_*i*_ based on the similarity of the items of the dictionary and the input feature *z*. Each wight *k*_*i*_ is computed via a softmax operation:


(3) $$\begin{array}{r} k_{i}=\frac{exp\left(d\left(z,m_{i}\right)\right)}{\sum_{j=1}^{N}exp\left(d\left(z,m_{i}\right)\right)}, \end{array}$$

where *d*(*z, m*_*i*_) denotes a correlation measurement between *z* and *m*_*i*_. Following the work, we define function of *d*(*z, m*_*i*_) as cosine similarity:


(4) $$\begin{array}{r} d\left(z,m_{i}\right)=\frac{z\cdot m_{i}^{T}}{\left||z||\cdot ||m_{i}|\right|}, \end{array}$$

Then, considering that the low-level features are more cluttered, some other group of ECG abnormalities may still have the chance to be reconstructed into the pacing ECG. To alleviate this issue, we apply a sparse coding strategy to promote the sparsity of *k*_*i*_. Sparse coding strategy encourages the model to represent a sample of pacing ECG using fewer but more relevant memory items, leading to learning more features from the memory unit. We define the sparse coding following the work Gong et al. (2019). Considering that all entries in *k* are non-negative, the sparse coding strategy is redefined via the continuous ReLU activation function as


(5) $$\begin{array}{r} \hat{k_{i}}=\frac{max\left(k_{i}-\alpha ,0\right)\cdot k_{i}}{|k_{i}-\alpha |+\epsilon }, \end{array}$$

where $\hat{k_{i}}$ represents the *i* − *th* entry in current memory state *k*, the *max*(*k*_*i*_ − α, 0) is also obtained as Relu activation. The α is a sparse threshold which is set the value in the interval [1/*N*, 3/*N*]. And ϵ is a very small positive scalar.

Finally, we normalize the weight vector $\hat{k}$ by letting ${\hat{k}}_{i}=k_{i}/||\hat{k}||$. Therefore, the output $\hat{z_{i}}$ of memory unit is defined as.


(6) $$\begin{array}{r} ẑ=\hat{k}\cdot D=\overset{N}{\underset{i=1}{\sum }}\hat{k_{i}}\cdot m_{i}, \end{array}$$

### 3.3. Training and Testing

Given a database *X* containing N samples, let $\hat{x_{i}}$ denote the reconstructed sample corresponding each input sample *x*_*i*_. In the training phase, the $L\left(x,\hat{x}\right)$ is used to measure the reconstruction error:


(7) $$\begin{array}{r} L\left(x,\hat{x}\right)=\frac{1}{n}\sum ||x_{i}-{\hat{x}}_{i}|{|}^{p}, \end{array}$$

where *p* is set to 1 or 2 in our paper. When *p* = 1, formula 7 is the mean absolute error, which can also be regarded as the L1-loss. When *p* = 2, formula 7 can be regarded as the mean square error, which is L2-loss. Due to the ECG abnormality diagnosis application scenario, we design ablation experiments to find the optimum value of *p*.

Due to the memory module of the testing phase, the learned memory content is fixed. Only the feature in the dictionary of the training type in the memory module can be retrieved for reconstruction. Thus the samples of one type can be reconstructed well. Conversely, the encoding of another ECG abnormality input will be replaced by the retrieved trained features, resulting in significant reconstruction error on this input data.

In the testing phase, we also need to define the rule to classify ECG abnormalities according to the MAE model. For example, given an input ECG data *x*_*i*_ and the reconstruction error are used to determine the classification. *P*_*n*_ denoted that the samples are the class of target domain *n*. Therefore, we define the set of samples in the target domain as:


(8) $$\begin{array}{l} T(x_{i})=\{x_{i}\in P_{n},with{\left\|x_{i}-f_{d_{n}}(f_{e_{n}}(x_{i};\theta_{e});\theta_{d})\right\|}^{p}\leq \|x_{i}\\ \;-{f_{d_{q}}(f_{e_{q}}(x_{i};\theta_{e});\theta_{d})\|}^{p}\}, \end{array}$$

where, the *f*_*e*_*n*__ and *f*_*d*_*n*__ represent the operation of encoder and decoder which the training phase records the latent features of target class *n* in the memory module. The *f*_*e*_*q*__ and the *f*_*d*_*q*__ represent the operation of encoder and decoder in which the training phase records the latent features of target class *q* in the memory module. This equation essentially defines the distance relationship between samples of the same classes and samples of different classes.

## 4. Experiments

In this section, we validate the proposed MAE framework for pacing ECG detection. Meanwhile, to show the applicability of the method, we also conduct experiments on the MIT-BIH Arrhythmia Database. First, the evaluation metric used in the experiments will be introduced. The quality and performance of the proposed MAE framework are evaluated by utilizing standard metrics: precision, recall, and f1-score. And then, the experimental database will be described. Finally, we present the experimental results and analysis. Additionally, the results are compared with other methods of ECG abnormality detection.

### 4.1. Evaluation Metric

In this paper, typical classification metrics, including precision, recall, and F1-score were used for each class. Precision is the ratio of the number of correct positive predictions to the total number of positive predictions. Recall is the ratio of the number of correct positive predictions to the total number of true positive and false negatives. F1-score is the weighted average of precision and recall. They are defined as:


(9) $$\begin{array}{r} Precision\left(+p\right)=\frac{TP}{TP+FP}, \end{array}$$

(10) $$\begin{array}{r} Recall\left(Rec\right)=\frac{TP}{TP+FN}, \end{array}$$

(11) $$\begin{array}{r} F1-score=\frac{2\times Precision\times Recall}{Precision+Recall}, \end{array}$$

where *TP*, *TN*, *FP*, and *FN* represent the numbers of true positives, true negatives, false positives, and false negatives, respectively.

### 4.2. Experiments on Pacing-ECG Detection

#### 4.2.1. The Pacing-ECG Database

The professional database plays a more important role in automatic ECG diagnosis than the algorithm and employed techniques. One of the obstacles in the research on fully automatic analysis in ECG is the insufficient quantity of available databases (Shen et al., 2020).

In this paper, we collect an extensive, novel ECG database named Pacing ECG Database. The pacing ECG database can be used to evaluate the proposed MAE framework. It should be noted that all extracted data were de-identified according to the privacy policy. Fully de-identified patient data can be used for research purposes. The main goal is to detect the morphology of pacing ECG from various interferences. And some of the samples may contain other abnormalities in the pacing ECG database. We aim to distinguish between the sample with a pacemaker and the sample without a pacemaker under the interference of these abnormalities.

**Figure 4:**
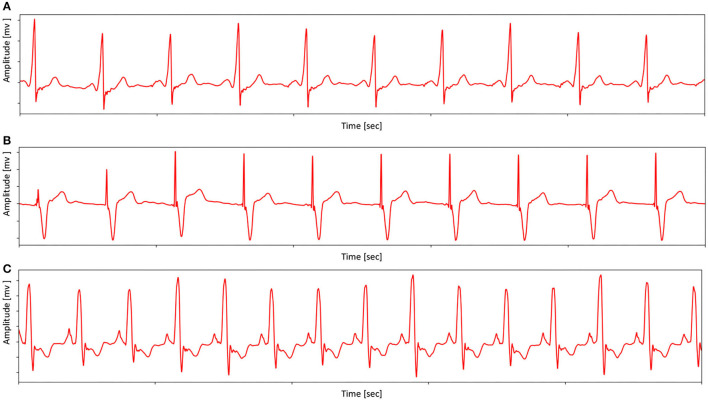
Examples ECG (from Lead II) in pacing ECG database. **(A)** An example shows the morphology of the pacing ECG. **(B)** An example shows that the morphology of pacing ECG receives other abnormal interference. **(C)** An example shows that the ECG morphology of the complete left bundle branch block is similar to pacing ECG. Note that sample **(A)** has no obvious pacemaker nail in lead II, but the proposed MAE method can distinguish the **(C)** and pacing type by other lead information.

The pacing ECG database contains 800 recordings of data annotated by the clinician lasting for 10 s, sampled at 500 Hz. Each sample acquired by the device is all 12-lead (channel) ECGs, digitized at 500 samples per second per channel and lasting for 10 s. The sample ECG sequences in the pacing ECG database are illustrated in Figure 4. The specific data distribution is shown in Table 1. We also evaluated the quality of ECG sequences. According to the standard of work (Shen et al., 2020), as shown in Table 2, each sample can be divided into good signal quality, medium signal quality, and poor signal quality. Our pacing ECG database only retains good quality signals.

**Table 1:** The specific data distribution in each database.

**Database**	**Number of patients**	**Record**	**Objective**
MIT-BIH (Moody and Mark, 2001)	48	N:90595, S:2781, V:7235, F:802, Q:8041.	Heartbeat arrhythmia analysis.
CPSC_2018 (Liu et al., 2018a)	6877	Normal:918, AF:1098, RBBB:1695, STD:825, PAC:556, PVC:672, LBBB:207, 1-VAB:704, STE:202.	Automatic identification of the rhythm abnormalities.
Pacing ECG	800	Pacing ECG:400, Routine ECG:400.	Pacing ECG detection.

**Table 2:** Specification for signal quality division (Shen et al., 2020).

**Category**	**Symbol**	**Definition**
Good	A	Signal with apparent P-QRS-T morphologies Signal with slightly baseline drift or transient artfacts
Medium	B	A good recording contaminated severely in a narrow window A good recording with one or a few missing signals A poor recording that may be interpretable with difficulty
Poor	C	Signal usefulness in clinical applications (maybe caused by misplaced electrodes, poor skin-electrode contact)

#### 4.2.2. Evaluation and Analysis

We carry out experiments on the pacing ECG database to further evaluate the proposed method for pacing ECG detection. The samples are split into training and test set with a rate of 3:1. Following the experimental setting used in these works (Gong et al., 2019; Hannun et al., 2019), the training set only consists of samples of the target class. There is no overlap between the training set and the testing set.

In this experiment, we mainly verify the effectiveness of our memory module and the encoder and decoder based on convolutional neural networks. First, we implement the encoder using 1-D convolution and the decoder using 1-D fractionally-strided convolution. Each convolutional layer is followed by batch normalization and a ReLU activation function. The details of the encoder and decoder are shown in Figure 3. We set the size of the memory module at 300. We also conduct the comparisons with some baseline variants of MAE to show the importance of the significant components, including the autoencoder without memory module (AE) and different reconstruction errors.

As shown in Table 3, we conduct several ablation studies to investigate the effectiveness of the major components of the proposed method, such as MAE and its backbone AE. And the MAE model with *P* = 2 gets better diagnosis results. As observed in Table 3, MAE with *P* = 2 achieve 91.8% F1-score, outperforming AE with *P* = 1 (4.0%, F1-score), AE with *P* = 2 (3.4% F1-score), MAE with *P* = 1 (1.7% F1-score). It is also seen that the proposed MAE achieves competitive results compared to other state-of-the-art methods. These methods like the Resnet backbone network architecture of Hannun et al. (2019) and a combination of RNN+CNN architecture of Yao et al. (2020). The MAE model outperforms the methods of the Hannun et al. (2019) by a margin, with a gain of 1.0% improvements in F1-score.

**Table 3:** The performance of +*P*, *Rec*, *F*1 − *score* of different methods on the pacing ECG database.

**Method**	**Pacing ECG database**		
	**+*P***	**Rec**	***F*1−*score***
Hannun et al., 2019	0.913	0.905	0.908
Oh et al., 2018	0.881	0.921	0.899
*AE*_*l*_1_	0.882	0.874	0.878
*AE*_*l*_2_	0.889	0.880	0.884
*MAE*_*l*_1_	0.906	0.897	0.901
*MAE*_*l*_2_	0.912	0.925	0.918

**Figure 5:**
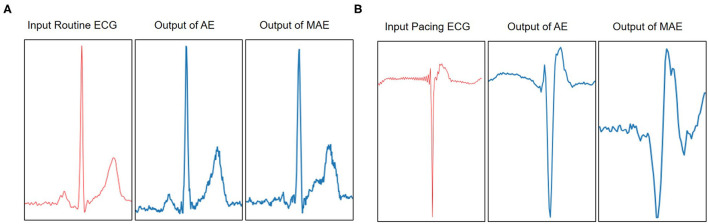
Visualization of the reconstruction results of AE and MAE on Pacing ECG. **(A)** The example model is trained on a type of pacing ECG. The input data is a routine ECG. Due to the periodicity of the ECG, the reconstruction data from the traditional AE model is also close to the input data. It is a failure case of ECG reconstruction in terms of error. But the MAE retrieves the training types memory items for reconstruction data and obtains the results significantly different from the input data types. The significant error between the reconstruction data and the input data means that the input data does not belong to pacing ECG. **(B)** The example model is trained on a type of routine ECG. The input data is a pacing ECG. The traditional AE model gets a failure case on the input data. But the MAE model obtains a higher significant error between reconstructions data and the input data. Therefore, it shows that the input data is a different type from the type of model.

**Figure 6:**
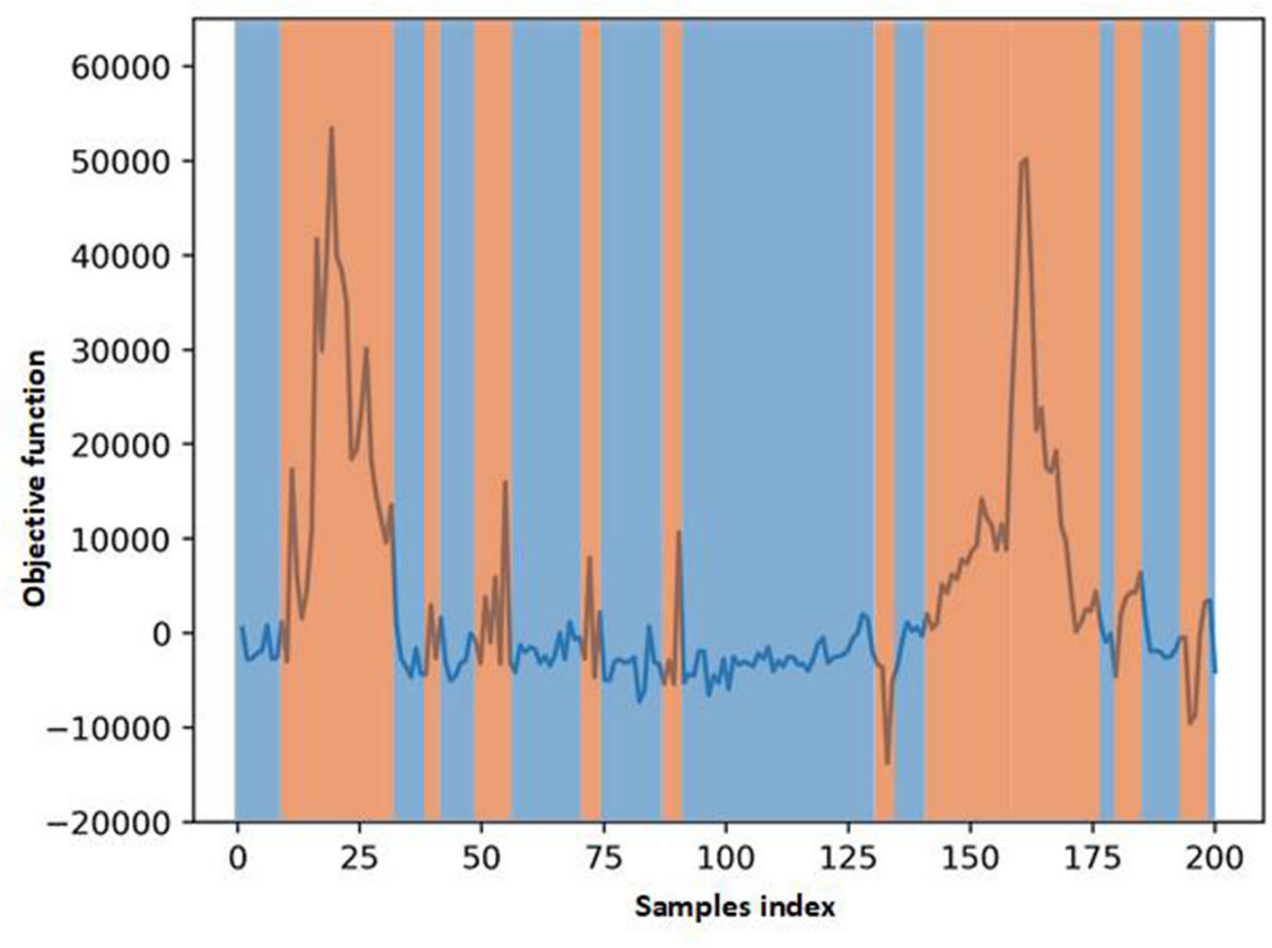
Scores of objective function obtain by MAE. The score changes immediately when some different types ECG samples in the test data.

In Figure 5, we visualize the ECG data reconstruction process under the memory module. Since the trained memory only records the latent features of training type, given a routine ECG sample as input, the MAE trained on pacing ECG type, resulting in significant reconstruction error in the kind of input data. Note that the reconstructed pacing ECG of MAE has a similar feature to the input routine ECG type because the memory module retrieves the most identical memory features. The ECG data has periodicity, and the AE model without memory module records some features that are more similar. Thus other types of samples sometimes may also be reconstructed well. Figure 6 shows that the objective function score obtained by MAE immediately changes when different types of ECG abnormalities samples appear in the dataset.

### 4.3. Experiments on MIT-BIH Arrhythmia Database

The proposed MAE model also can be generally applied to diagnose other ECG abnormalities. We carry out the experiments on the MIT-BIH Arrhythmia database to evaluate the proposed method.

#### 4.3.1. MIT-BIH Arrhythmia Database

**Figure 7:**
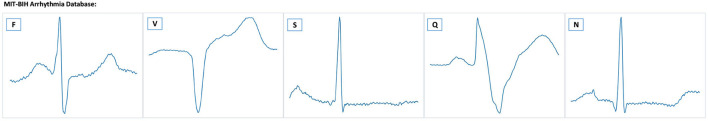
Example ECG signals from the MIT-BIH database. There are five heartbeat classes named N, S, V, F, and Q. The meaning of each letter is shown in Table 4.

The MIT-BIH Arrhythmia database (Moody and Mark, 2001) contains 48 half-hour excerpts of two-channel ambulatory ECG recordings. The recordings are digitized at 360 samples per second per channel. Each record comprises two signals. For all the records, the first one is the modified-lead II (MLII), whereas the second one corresponds to V1, V2, V4, or V5, depending on the records. Therefore, only the MLII is provided by all the records. The database contains two or more expert cardiologists independently annotated approximately 110,000 beats, all of them, and the disagreements were resolved. The MIT-BIH heartbeat types are grouped into five heartbeat classes which are recommended by the Association for the Advancement of Medical Instruments (AAMI), as shown in Table 4. Example signals for the MIT-BIH Arrhythmia database are shown in Figure 7.

**Table 4:** Mapping the heartbeat types to the AAMI heartbeat categories and data distribution statistics (Moody and Mark, 2001).

**AAMI**	**MIT-BIH**	**Heartbeat types**	**Total**
N	N	Normal beat	90,462
	L	Left bundle branch block beat	
	R	Right bundle branch block beat	
	e	Atrial escape beat	
	j	Nodal(Junctional) escape beat	
S	A	Atrial premature beat	2,777
	a	Aberrated atrial premature beat	
	J	Nodal(Junctional) premature beat	
	S	Supraventricular premature or ectopic beat	
V	V	Premature ventricular contraction	7,223
	E	Ventricular escape beat	
F	F	Fusion of ventricular and normal beat	802
Q	/	Paced beat	8,027
	f	Fusion of paced and normal beat	
	Q	Unclassifiable beat	

#### 4.3.2. Evaluation and Analysis

**Figure 8:**
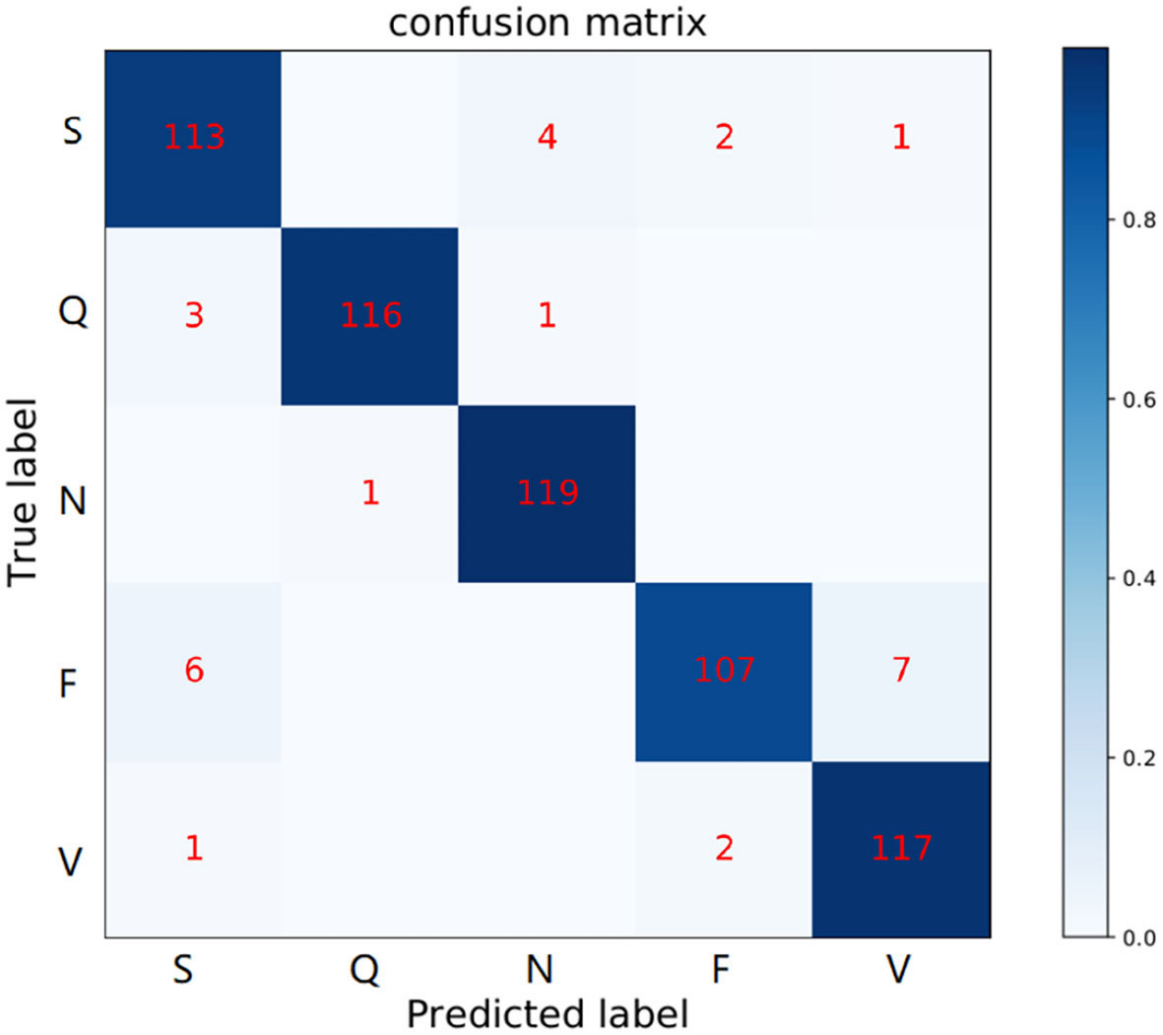
Confusion matrix of MAE model in the MIT-BIH Arrhythmia database. The row labels represent the true class records in each row and the column labels represent the class records predicted by our method. Numbers in each grid show the number of records classified as column labels when its true class is indicated by row label.

It should be noted that the MIT-BIH Arrhythmia database has unbalanced distribution. Therefore, we abandon some samples, which doesn't affect the final performance. Following the works (Kachuee et al., 2018; Wang et al., 2021), we balanced the number of beats in each type before splitting the testing phase. This paper selects and tests on 600 heartbeat samples. In this paper, only lead II is considered to detect the ECG abnormalities. This decision is motivated by the following works (Mondéjar-Guerra et al., 2019), which proved that using only one lead is sufficient for the arrhythmia classification task. And the 235 points are extracted as single heartbeat morphology features. For experiment settings, the total number of iterations is 200, and the batch size is set to 16. We apply an initial learning rate of 10^−5^. The Figure 8 shows the classification results of the MAE model with data splitting. In addition, among those works shown in Table 5, these methods are designed for improving the accuracy of ECG heartbeat abnormality detection. Nevertheless, the MAE model can still exceed them with a large margin, which can further demonstrate the effectiveness of the proposed approach for ECG heartbeat abnormality detection. Meanwhile, MAE produces the highest F1-score for most heartbeat types, such as type of F performance has significant improvement. The improvements are mainly attributed to the memory module.

**Table 5:** Comparisons of +*P*, *Rec*, and *F*1 − *score* for each type on the MIT-BIH Arrhythmia database.

**Methods**	**N**			**S**			**V**			**F**			**Q**		
	**+P**	**Rec**	**F1**	**+P**	**Rec**	**F1**	**+P**	**Rec**	**F1**	**+P**	**Rec**	**F1**	**+P**	**Rec**	**F1**
Zhang et al., 2014	0.990	0.889	0.937	0.359	0.791	0.494	0.927	0.855	0.842	0.137	0.938	0.220	–	–	–
Ming et al., 2020	0.984	0.953	0.968	0.938	0.785	0.832	0.938	0.957	0.947	0.088	0.438	0.146	–	–	–
Li et al., 2019	0.975	0.910	0.941	0.780	0.638	0.702	0.865	0.884	0.874	0.907	0.873	0.890	0.994	0.966	0.994
Hannun et al., 2019	0.948	0.950	0.948	0.826	0.720	0.769	0.872	0.890	0.880	0.942	0.786	0.856	0.990	0.990	0.990
Mondéjar-Guerra et al., 2019	0.982	0.959	0.970	0.497	0.781	0.607	0.939	0.947	0.994	0.236	0.124	0.162	–	–	–
Memory-AE	0.960	0.992	0.972	0.936	0.867	0.900	0.807	0.975	0.883	0.964	0.892	0.926	0.991	0.975	0.983

Specifically, Table 5 shows the +*p* and *Rec* and F1 of the proposed MAE model and other popular methods on the database testing set. The experimental results show that the F1-score of N category is 97.2%, the F1-score of S category is 90.0%, the F1-score of V category is 88.3%, the F1-score of F category is 92.6%, and the F1-score of Q category is 98.3%. Table 6 shows the overall results of the MAE model and compares it with the state-of-the-art methods in other literature. Some method results show that the classification performances for type F and type Q are not satisfactory. It may be that these beats are harder to classify. On the whole, our MAE model achieves better performance for type N, S, V, F, and Q. Moreover, the MAE model can also outperform these typical classifiers based on CNN, for example, the 9-layer CNN proposed by Acharya et al. (2017) (92.5%, 2.4% higher F1-score), the combination of CNN with LSTM proposed by Shi et al. (2019) (93.6%, 1.3% higher F1-score).

**Table 6:** Comparisons with the popular methods for overall types on the MIT-BIH Arrhythmia database.

**Typical methods**	**ECG beat types**	**Classifier**	**Performance**		
			**+P**	**Rec**	**F1-score**
Acharya et al., 2017	N S V F Q	CNN	89.5%	95.9%	92.5%
Niu et al., 2020	N S V F Q	Multi-Perspective CNN	96.4%	–	–
Shi et al., 2019	N S V F	CNN-LSTM	94.2%	93.1%	93.6%
Mondéjar-Guerra et al., 2019	N S V F	Ensemble SVMs	94.5%	70.3%	80.6%
Proposed MAE	N S V F Q	Memory-based Autoencoder	95.8%	94.0%	94.9%

**Figure 9:**
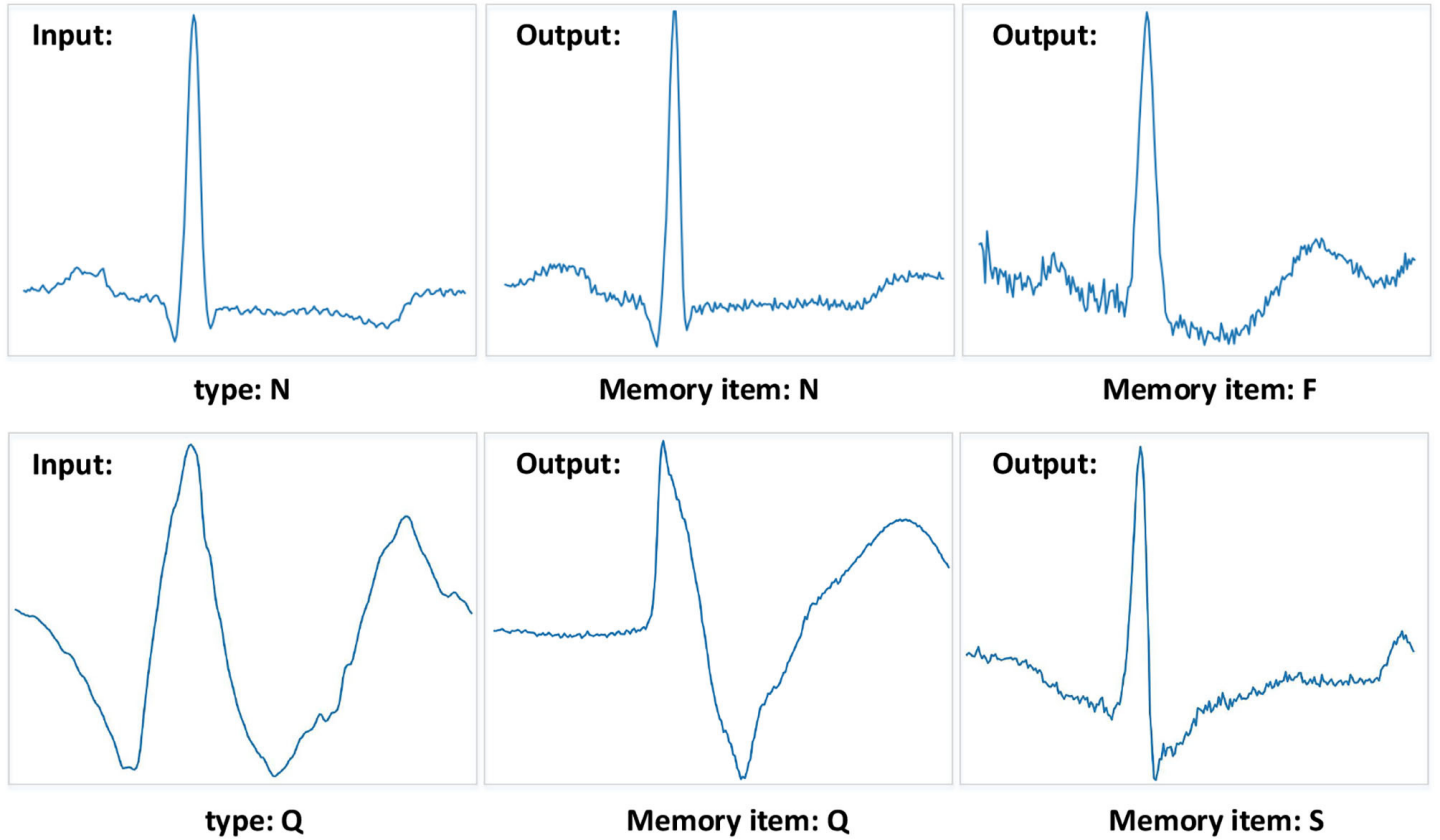
Examples of the MIT-BIH Arrhythmia database. The “N” type and “Q” type are set to the input samples. And the different memory items mean that the training phase records corresponding types of features. The MAE model can get a significant reconstruction error when the input data is different from the recorded data type.

We also visualize data reconstruction in the MIT-BIH Arrhythmia database, shown as Figure 9. The trained memory module records the latent features of the input type. Given a testing data ECG of “N” type, the memory module trained on “N” type reconstructs the “N” type, resulting in a low error on the input data. But the memory items trained on the “F” type reconstruct the “N” type, given a testing data ECG “N” type, resulting in a significant error on the input data “N” type. Note that the reconstructed “F” type of MAE has a similar shape to the input “N” since the memory module retrieves the most similar latent features. By comparing the errors, we can easily get the type of input test data. Despite some data having noise, the MAE model can still detect the type of heartbeat, which benefits from the memory module designed in MAE. Meanwhile, the compelling performance also demonstrates the generalization ability of the MAE model.

## 5. Conclusion and Future work

In this paper, a memory-based autoencoder was proposed to construct the intelligent diagnosis model for ECG abnormality detection. We designed a novel autoencoder using a memory module to record the latent features from the training data of corresponding types. The key features of MAE are to preserve the latent features to obtain low average reconstruction error in the training phase. And in the testing phase, the output data of reconstruction will reference the memory items which were selected as similar items of the encoding of the input data. Furthermore, we also defined an objective function that can compute the distance relationship between samples of the same types and samples of different types. In brief, the proposed MAE can well reconstruct the input data consistent with the training types to get a low objective function error and enlarge the objective function error of other abnormal ECG types, which the objective function is the better criterion of abnormal ECG detection. The results demonstrate that the proposed model achieves a significant performance gain with accuracy, sensitivity, and F1-score through a series of experiments.

In future work, since the same types of ECG abnormality has differences between different patients, we aim to record the latent features in the same types of ECG abnormality of other individuals. By analyzing these individual differences, we further explore to improve the accuracy, sensitivity, and F1 score of the model. Additionally, the diagnostic efficiency of the model is also an important indicator of clinical ECG diagnosis. We also aim to design a more lightweight and efficient diagnostic model which can be better applied in clinical ECG diagnosis.

## Data Availability Statement

The raw data supporting the conclusions of this article will be made available by the authors, without undue reservation. The publicly released dataset of the MIT-BIH Arrhythmia Database is available at: https://www.physionet.org/content/mitdb.

## Ethics Statement

The studies involving human participants were reviewed and approved by Life Science Ethics Review Committee Zhengzhou University. Written informed consent for participation was not required for this study in accordance with the national legislation and the institutional requirements.

## Author Contributions

ZT and ZG conceived the study. ZG performed the design and implementation of the work and wrote the manuscript. HC and ZT helped with the experiment design and implementation. LY provided the experimental data and annotated the data. BZ and ZW helped to review and improved the manuscript. All authors read and approved the final manuscript.

## Conflict of Interest

The authors declare that the research was conducted in the absence of any commercial or financial relationships that could be construed as a potential conflict of interest.

## Publisher's Note

All claims expressed in this article are solely those of the authors and do not necessarily represent those of their affiliated organizations, or those of the publisher, the editors and the reviewers. Any product that may be evaluated in this article, or claim that may be made by its manufacturer, is not guaranteed or endorsed by the publisher.
